# Overexpression of *SoCYP85A1* Increases the Accumulation of Castasterone and Confers Enhanced Black Shank Tolerance in Tobacco Through Modulation of the Antioxidant Enzymes’ Activities

**DOI:** 10.3389/fpls.2019.00349

**Published:** 2019-03-22

**Authors:** Fangmeng Duan, Wenwen Song

**Affiliations:** The Key Laboratory of Integrated Crop Pest Management of Shandong Province, College of Plant Health and Medicine, Qingdao Agricultural University, Qingdao, China

**Keywords:** black shank disease, transgenic tobacco, *SoCYP85A1*, castasterone, defense enzymes

## Abstract

Black shank caused by *Phytophthora nicotianae* is one of the most devastating diseases in tobacco production. In this study, we characterized a novel cytochromic resistance gene, *SoCYP85A1*, from spinach, which was upregulated in response to *P. nicotianae* infection. Overexpression of *SoCYP85A1* in tobacco resulted in remarkable resistance to pathogen inoculation, with diverse resistance levels in different transgenic lines. Meanwhile, a significant accumulation of castasterone (CS) was detected in transgenic plants when challenged with the pathogen. Moreover, activities of antioxidant enzymes were enhanced by *SoCYP85A1* in the transgenic lines as compared to those in the wild types inoculated with *P. nicotianae*. In addition, the alteration of CS content resulted in interference of phytohormone homeostasis. Overall, these results demonstrate that *SoCYP85A1* can participate in the defense response to *P. nicotianae* through the involvement of defense enzymes and by interaction with certain phytohormones. Our findings suggest that *SoCYP85A1* could be used as a potential candidate gene for improving resistance to black shank disease in tobacco and other economic crops.

## Introduction

Members of oomycetes are destructive pathogens that cause damages to important economic crops (Kamoun et al., 2015). Among the oomycetes that are pathogenic to plants, *Phytophthora nicotianae* is one of the most devastating for tobacco (*Nicotiana tabacum* L.) production (Gallup et al., 2018); it causes serious disease in the roots and stems of tobacco in many tobacco-growing regions around the world (Johnson et al., 2002). Plants have evolved defense mechanisms to resist pathogen infections. One such mechanism involves regulation of the biosynthesis of phytohormones, such as abscisic acid (ABA), gibberellic acid (GA), jasmonic acid (JA), and salicylic acid (SA) (Vlot et al., 2009; Fang et al., 2016; Gao et al., 2016; Tang et al., 2017).

Brassinosteroids (BRs) are one of the most important phytohormones and involve in various physiological processes (Choudhary et al., 2012). In addition of their roles in plant growth and development (Arteca and Arteca, 2001; Yu et al., 2004; Gruszka, 2013), BRs have been implicated in tolerance to pathogens (Nakashita et al., 2003; Albrecht et al., 2012). However, most of the previous studies focusing on the regulation of plant response to pathogens by BRs have been performed using exogenously applied BRs (Nakashita et al., 2003; Divi et al., 2010; Albrecht et al., 2012). The genetic mechanisms underlying the role of endogenous BRs in regulating the response of plants to pathogens remain poorly understood.

Over the past few years, the biosynthesis pathway of BRs has been elucidated, and key enzymes involved in the pathway have been identified (Shimada et al., 2003; Kim et al., 2005; Ohnishi et al., 2012). Among these, CYP85A1 catalyzes the conversion of intermediate metabolites to castasterone (CS) (Shimada et al., 2001), which is the precursor of brassinolide (BL), the end-product of BRs biosynthesis pathway. However, the rate of conversion of CS to BL is extremely low (Noguchi et al., 2000). CS possesses its own function and performs the same biological activity as BL (Kim et al., 2005). In a previous work, we observed that overexpression of *SoCYP85A1* (GenBank Accession: KT900949), a spinach cytochrome P450 enhanced drought tolerance in transgenic tobacco by increasing the CS content. CS was assumed to be a bioactive BR and displayed the same biological function (Duan et al., 2017). However, whether endogenous CS participates in plant response to pathogen infection remains unknown.

To explore the function CS in plants subjected to pathogen invasion, we analyzed tobacco seedlings overexpressing *SoCYP85A1* infected with *P. nicotianae*. It was observed that overexpression of *SoCYP85A1* enhanced black shank tolerance of transgenic tobacco seedlings compared to that of wild type seedlings. Furthermore, this enhanced tolerance was demonstrated to be through the upregulation of activities of defense enzymes at the transcriptional level. In addition, CS was identified as a bioactive component in tobacco, which was induced by the *P. nicotianae* infection. The alteration of CS content resulted in interference of phytohormone homeostasis. This study provides new information for the disease control network and a new strategy for disease resistance breeding.

## Materials and Methods

### Plant Materials and Growth Conditions

The genotype of tobacco that was genetically engineered was *N. tabacum* cv. Xanthi-nc. T_3_ seeds from the transgenic lines overexpressing *SoCYP85A1* driven by the CAMV35S promoter were produced and confirmed as described in our previous study (Duan et al., 2017), in which the *SoCYP85A1* gene has been stable inheritance and expression. T_3_ seeds were grown and developed to T_4_ transgenic soybean plants for further analysis. The wild type (WT) and transgenic lines were grown in growth chambers under a photoperiod of 16 h light/8 h dark and maintained at 30°C and 60% relative humidity for 1 month.

### Genomic PCR and GFP Analysis of Transgenic Plants

Genomic DNA was extracted from tobacco leaves using Plant DNA Kit (Omega Bio-tek, Norcross, GA, United States). The primers (Forward: 5′-ATGGCCGTTTTTATGGTGGTTTTTGCTGT-3′ and Reverse: 5′-CTAATAACTCGAAACTCGAATGC-3′) were used to amplify the open reading frame (ORF) of the *SoCYP85A1* gene. For green fluorescent protein (GFP) analysis, fluorescence signal was detected with the NEWTON 7.0 smart imaging system (Vilber Lourmat, France).

### Stress Treatments

For *P. nicotianae* treatment, 10 seedlings per type in each treatment were infected with the pathogen following the method described by Gao et al. (2010). The plants treated with the same amount of sterile water in the same way served as controls. The tested seedlings were transplanted in another growth chamber under a 16 h light/8 h dark cycle at 35°C and 90% relative humidity. Each treatment was performed in three biological replicates, in which there were three technical replicates.

### Assays of Resistance of Transgenic Tobacco Against *P. nicotianae*

The disease symptoms on each seedling were monitored at 2, 3, 4, and 7 days post inoculation (dpi), respectively. The severity of disease was graded according to GB/T 23222-2008 (National Standardization Management Committee, 2009). Disease index (DI) was calculated using the following formula: DI (%) = [Σ (DG_i_ × n_i_)/(DG_i max_ ×*n*)] × 100, where DG_i_ is the value of the disease grade, *ni* is the number of plants with each disease grade, DG_i max_ is the maximum value of disease grades, and *n* is the total number of inoculated plants. The unifoliate leaves were harvested at 0 and 4 dpi, immediately frozen in liquid nitrogen, and stored at -80°C to investigate the function of *SoCYP85A1*.

### Quantitative Real-Time RT-PCR of *SoCYP85A1*

Total RNA was extracted from WT and transgenic tobacco seedlings with Plant RNA Kit (Omega Bio-tek, Norcross, GA, United States). Reverse transcription was performed using Prime Script^TM^ RTase Kit (TaKaRa, Dalian, China) in accordance with the manufacturer’s instructions. The relative expression level of *SoCYP85A1* was examined by SYBR *Premix Ex Taq* II Kit (TaKaRa, Dalian, China) before and after the inoculation of *P. nicotianae*. The PCR program was as follows: 95°C for 5 min followed by 40 cycles of 30 s at 95°C, 30 s at 60°C, and 30 s at 72°C. *Spinacia oleracea* actin gene (GenBank: JN987183.1) served as an internal control to determine the relative transcript levels of *SoCYP85A1*. The relative gene expression level was calculated using the ΔΔCt method (Livaka and Schmittgenb, 2001). qRT-PCR was performed in three biological replicates and each reaction was proceeded three times.

### Extraction and Quantification of Endogenous BRs

Both extraction and quantification of endogenous BRs were conducted according to Ding et al. (2013).

### Measurement of the Activities of Antioxidant Enzymes and Their Corresponding Gene Expression Levels

The activities of defense-related enzymes, including superoxide dismutase (SOD), peroxidase (POD), polyphenol oxidase (PPO), and catalase (CAT), were spectrophotometrically detected using different kits (obtained from Jiancheng, Nanjing, China; catalog number A001-4 for SOD, A084-3 for POD, A136 for PPO, and A003-3 for CAT) in accordance with the manufacturer’s instructions.

### qRT-PCR Analysis of Defense-Related Genes

qRT-PCR was employed to examine the relative expression levels of defense-related genes, which included four genes encoding the enzymes mentioned above (*NtSOD*, *NtAPX*, *NtPPO*, and *NtCAT*), ABA biosynthesis gene (*NtNCED1*), SA-responsive genes (*NtPR1a/c* and *NtPR2*), JA-responsive gene (*NtPR1b*), and HR-associated gene (*NtHSR203J*). The sequences of the all the primers used for analysis were listed in Table 1. The *NtActin* gene served as the internal control. The analysis was performed for three biological replicates of each sample.

**Table 1:** Specific primers designed for qRT-PCR.

Genes	Forward primer sequence (5′-3′)	Reverse primer sequence (5′-3′)
*SoCYP85A1*	AATCAAGCTCAACTGCCCAAC	CAGGGAGGTCAATAGGGAGAGA
*SoActin*	GATTCTGGTGATGGTGTTAGT	CTCCGATTGTGATGACTTGT
*NtSOD*	CCGTCGCCAAATTGCATAG	CGATAGCCCAACCAAGAGAAC
*NtAPX*	CAAATGTAAGAGGAAACTCAGAGGA	AGCAACAACTCCAGCTAATTGATAG
*NtPPO*	TGATAACTAATGCTCCTTGC	CTCCGAGTTCAACCAATCT
*NtCAT*	AGGTACCGCTCATTCACACC	AAGCAAGCTTTTGACCCAGA
*NtNCED1*	CTATTTCCACTTCAAAACCAACCAC	GGCACTTTCCACGGCATCT
*NtPR1a/c*	AACCTTTGACCTGGGACGAC	GCACATCCAACACGAACCGA
*NtPR2*	TGATGCCCTTTTGGATTCTATG	AGTTCCTGCCCCGCTTT
*NtPR1b*	AACCCATCCATACTATTCCTTG	GCCGCTAACCTATTGTCCC
*NtHSR203J*	AGGAAGTATCCGGCTGGCTTAGA	GAAGTAGTCATGGGGTGGGACTG
*NtActin*	CAAGGAAATCACCGCTTTGG	AAGGGATGCGAGGATGGA


### Extraction and Quantification of Endogenous ABA, GA3, JA, and SA

Extraction and quantification of ABA, GA3, and JA were performed as described previously (Duan et al., 2017). The procedure for the measurement of SA was slightly modified from the method described Duan et al. (2017). The extract of SA was vacuum evaporated to remove the solvent at 40°C and dissolved in 0.5 mL Reagent 3 for HPLC analysis. The flowing phase was detected at a wave-length of 240 nm for 30 min. Each sample was monitored using HPLC (e2695, Waters, United States) equipped with a reversed phase Kromasil C18 column.

### Statistical Analysis

The experimental design was a completely randomized block design with three replicates. Each replicate contained three plants. Statistical comparisons were implemented using the SPSS statistical software (ver.16.0, SPSS Inc., Chicago, IL, United States) by one-way analysis of variance. A Tukey’s test was conducted to evaluate treatment efforts. Error bars represent standard deviations (SD) with different letters at *P* < 0.05 significant level.

## Results

### Molecular Confirmation of Transgenic Tobacco Plants Overexpressing *SoCYP85A1*

**FIGURE 1:**
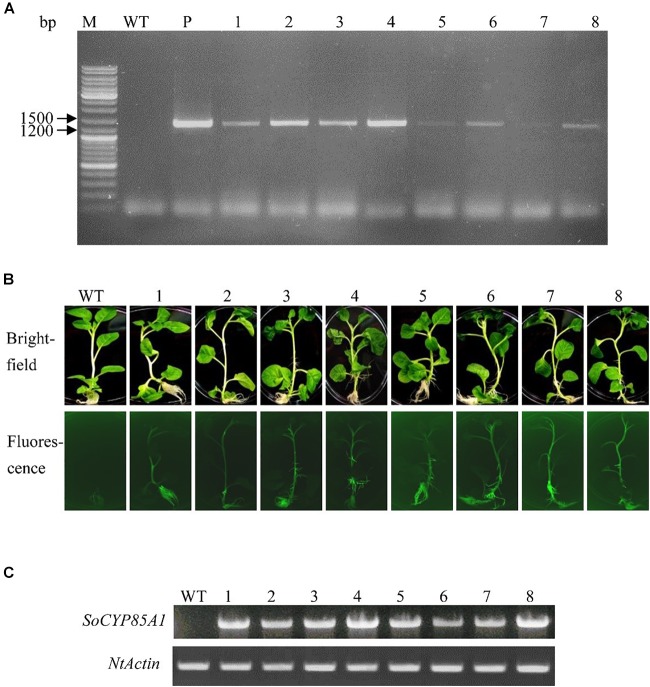
Molecular identification of transgenic tobacco lines. **(A)** PCR analysis of genomic DNA with specific primers for the ORF of *SoCYP85A1*. **(B)** GFP analysis of transgenic lines. **(C)** RT-PCR analysis of WT and different transgenic lines. M, Molecular marker; WT, Wild type; P, Plasmid; 1–8: transgenic lines.

The *SoCYP85A1* gene was inserted in pB7WG2D,1-GFP to create the overexpressing vector. T_4_ generation of transgenic tobacco plants was confirmed by PCR analysis (Figure 1A) and fluorescence detection of GFP protein (Figure 1B). The 1,392-bp fragment of the *SoCYP85A1* gene was amplified in the transgenic lines. Further analysis by semi-quantitative RT-PCR confirmed successful integration of *SoCYP85A1* in all the tested transgenic lines (Figure 1C), among which, the transgenic line 8 (L8) had the highest level of the overexpressed transcript.

### Overexpression of *SoCYP85A1* Enhances the Resistance of Tobacco Plants to *P. nicotianae*

**FIGURE 2:**
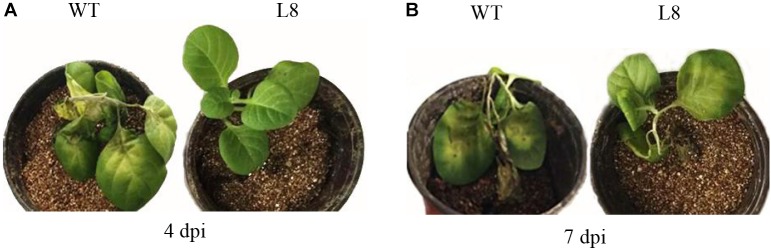
Tobacco plants inoculated with *Phytopthora nicotianae.* **(A)** Phenotypes of wild-type (WT) and L8 line infected with *P. nicotianae* at 4 days post inoculation (dpi) and **(B)** 7 dpi, respectively.

To investigate whether the overexpression of *SoCYP85A1* could enhance the resistance to *P. nicotianae*, the T_4_ transgenic tobacco plants were inoculated with *P. nicotianae*. All the transgenic lines exhibited enhanced but different levels of resistance to the pathogen (Table 2). Among the eight transgenic lines, L4 and L8 possessed higher resistance, with both lower disease rate and disease index, whereas the stem of WT plants displayed severe necrosis, with 7.23% disease index and 75.56% rate at 7 dpi. As shown in Figure 2A, the infection of pathogen resulted in a slightly necrotic stem and wilting of leaves in the WT plants, whereas no symptoms were observed at 4 dpi in the L8 line, which accumulated the highest level of the *SoCYP85A1* transcript. Extremely severe wilting, necrosis, and lodging symptoms appeared in the WT seedlings at 7 dpi, whereas only faint wilting symptoms were observed in L8 plants (Figure 2B). The above results indicated that the overexpression of *SoCYP85A1* notably enhanced the resistance against *P. nicotianae* in transgenic tobacco plants. Based on the results, L4 and L8 were selected as the representative lines for further analyses.

**Table 2:** Disease rate and disease index of wild-type (WT) and different transgenic lines after inoculation with *Phytopthora nicotianae*.

Transgenic lines	Disease rate (%)	Disease index (%)
WT	75.56 ± 8.39 c	7.23 ± 0.96 e
L1	32.22 ± 3.94 ab	5.01 ± 0.22 cd
L2	43.33 ± 6.67 b	5.52 ± 0.75 de
L3	27.78 ± 6.94 ab	3.42 ± 0.52 abc
L4	20.89 ± 6.94 ab	2.71 ± 0.62 bc
L8	30.00 ± 5.77 ab	4.69 ± 0.60 bcd
L6	28.89 ± 5.09 ab	3.30 ± 1.34 abc
L7	25.56 ± 4.09 ab	2.75 ± 0.54 ab
L8	15.56 ± 2.93 a	1.43 ± 0.33 a


*Different capital letters indicate significant differences at *P* < 0.05.*

### Overexpression of *SoCYP85A1* Increases the Accumulation of *SoCYP85A1* Transcript and CS After Pathogen Inoculation

**FIGURE 3:**
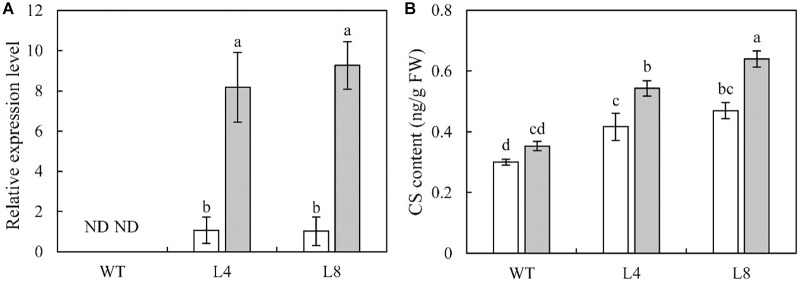
Effect on castasterone (CS) content in wild-type (WT), and transgenic lines 4 and 8 inoculated with *P. nicotianae*. **(A)** Expression level of *SoCYP85A1* and **(B)** CS content in WT and transgenic lines 4 and 8 before and after infection with *P. nicotianae* at 4 days post inoculation (dpi). Different capital letters indicate significant differences at P < 0.05.

The qRT-PCR results showed that the transcription of *SoCYP85A1* was not detectable in the un-infected or infected WT plants. In contrast, pathogen infection induced a significant increase in the transcript level of *SoCYP85A1* in the transgenic lines (Figure 3A).

To understand whether *SoCYP85A1* indeed functions in BRs biosynthesis, the endogenous BRs in the WT and transgenic plants were examined before and after the inoculation of the pathogen. The intermediates in BR biosynthetic pathway, which include teasterone (TE), typhasterol (TY), 6-deoxo castasterone (6-deoxoCS), and 28-norcastasterone (28-norCS) were undetectable in both WT and transgenic lines under the absence and presence of the pathogen. No significant changes were observed in the content of BL. However, compared to WT plants, an obvious increase in the content of CS was detected in L4 and L8 plants. Furthermore, when subjected to pathogen infection, WT plants showed a slight increase in the content of CS, whereas L4 and L8 showed a remarkable increase (Figure 3B). These results suggested that overexpression of *SoCYP85A1* enhanced the content of CS in the transgenic tobacco plants. Moreover, pathogen infection resulted in a significant increase in CS content in the transgenic plants.

### Overexpression of *SoCYP85A1* Reduces the Accumulation of ABA, GA3, JA, and SA After *P. nicotianae* Infection

**FIGURE 4:**
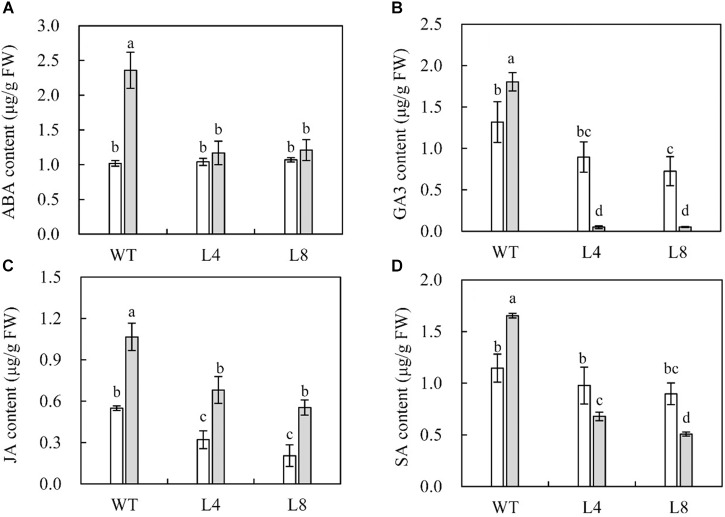
Contents of endogenous **(A)** abscisic acid (ABA), **(B)** gibberellic acid (GA3), **(C)** jasmonic acid (JA), and **(D)** salicylic acid (SA) in wild-type (WT) and transgenic plants under control and *P. nicotianae* inoculation conditions at 4 days post inoculation (dpi). Different capital letters indicate significant differences at P < 0.05.

To further explore the potential function of *SoCYP85A1* upon *P. nicotianae* infection, HPLC analysis of leaf extracts from WT and transgenic plants was performed to quantify the endogenous contents of ABA, GA3, JA, and SA. No significant differences in the contents of the phytohormones were detected between the WT and transgenic seedlings before pathogen treatment, with the exception of SA, whose accumulation was much higher in WT than in the transgenic lines (Figure 4D). Significantly, WT plants had higher contents of the four phytohormones compared to those in transgenic plants after pathogen infection. It was worth noting that the contents of GA3 and SA decreased sharply in L4 and L8 lines after the pathogen attack (Figure 4B,D). Conversely, transgenic plants overexpressing *SoCYP85A1* showed a higher accumulation of JA upon pathogen infection (Figure 4C). However, the ABA content was increased slightly in the transgenic seedlings. These results highlighted that alteration of CS content could alter the abundance of other phytohormones.

### Overexpression of *SoCYP85A1* Increases the Activities of Some Defense Enzymes After *P. nicotianae* Infection

**FIGURE 5:**
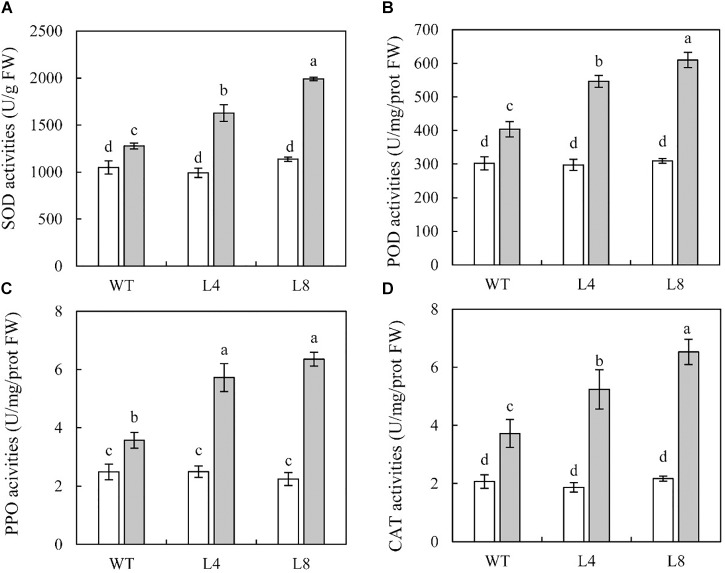
Activities of defense-related enzymes in wild-type (WT) and transgenic lines inoculated with *P. nicotianae* at 4 days post inoculation (dpi). **(A)** superoxide dismutase (SOD), **(B)** peroxidase (POD), **(C)** polyphenol oxidase (PPO), and **(D)** catalase (CAT). Different capital letters indicate significant differences at P < 0.05.

To examine whether *SoCYP85A1* overexpression would cause physiological changes in transgenic lines, the activities of four key defense enzymes (SOD, POD, PPO, and CAT) were determined in the WT and transgenic plants with and without pathogen inoculation. The results demonstrated that before inoculation the activities of these enzymes were not much different between the WT and the two transgenic lines (Figure 5). However, when exposed to the pathogen, all the treated seedlings displayed an obvious increase in the activities of SOD, POD, PPO, and CAT, which were significantly higher in L8, showing 58, 51, 78, and 76% increase, respectively, compared to those in the WT seedlings (Figure 5). These results demonstrated that overexpression of *SoCYP85A1* could enhance the activities of defense enzymes in transgenic seedlings upon challenge with *P. nicotianae*.

### Overexpression of *SoCYP85A1* Increases the Transcript Levels of Genes Encoding Defense Enzymes After *P. nicotianae* Infection

**FIGURE 6:**
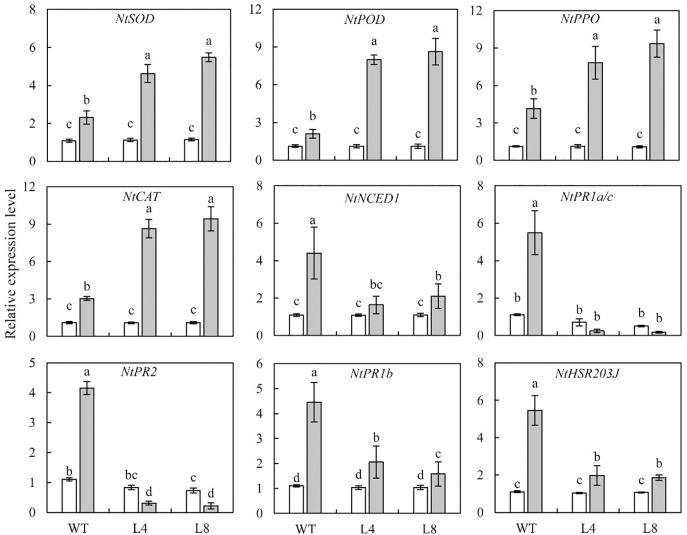
Relative transcript levels of genes involved in the resistance of wild-type (WT) and transgenic lines to *P. nicotianae* infection at 4 days post inoculation (dpi). Different capital letters indicate significant differences at P < 0.05.

For deciphering the molecular mechanism through which the overexpression of *SoCYP85A1* could enhance the resistance to *P. nicotianae*, qRT-PCR analysis was carried out to detect the transcript levels of genes in both WT and transgenic plants before and after the pathogen infection. In the absence of pathogen infection, no obviously differences were detected in the transcript levels of genes in WT and transgenic seedlings, with the exception of *NtPR2*, which exhibited a slight or significant decrease in L4 and L8 lines, respectively (Figure 6). However, when subjected to pathogen treatment, the transcript levels of *NtSOD*, *NtAPX*, *NtPPO*, and *NtCAT* were notably enhanced in transgenic plants compared to those in the WT plants, while the transcript levels of *NtNCED1*, *NtPR1b*, and *NtHSR203J* were lower in the transgenic lines than those in the WT plants (Figure 6). Pathogen infection resulted in a decrease in the abundance of *NtPR1a/c* and *NtPR2* transcripts in both the L4 and L8 lines. This result, together with those mentioned above, demonstrate that overexpression of *SoCYP85A1* could enhance the resistance of tobacco plants to *P. nicotianae* infection through upregulation of the expression of genes encoding defense enzymes at the transcriptional level.

## Discussion

In previous studies, the effects of BRs on plants were mostly investigated using exogenous application of BRs (Nemhauser et al., 2006). Thus, the role of the endogenous BRs in the regulation of plant diseases remained elusive and required further experimentation. Although the function of endogenous BRs in the growth and development of plants was amply highlighted in previous research (Pérez-España et al., 2011; Li et al., 2016a,b), their involvement in plant’s response to pathogen invasion needed to be verified. Here, we provided evidence that an increase in the content of endogenous BRs could enhance the resistance of tobacco plants overexpressing *SoCYP85A1* to *P. nicotianae* by enhancing the activities of defense enzymes. Our results provided a potential approach for enhancing black shank tolerance of tobacco plants through the regulation of genes involving in the biosynthetic pathway of BRs.

### *SoCYP85A1* Was First Identified to Enhance the Resistance to *P. nicotianae* in Tobacco Transformants

Over the past few years, molecular biology studies have been conducted to investigate the interaction of tobacco plants with *P. nicotianae*. Lipoxygenase (LOX) was the first proved enzyme during this interaction. The expression of antisense LOX gene could suppress the incompatible interaction between tobacco and this pathogen, which suggested the crucial role of LOX in the defense of host plants (Rancé et al., 1998). Subsequently, several defense-related genes had been identified to play a role in the response to black shank disease in tobacco, among which, the expressions of glutathione S transferase (GST), hsr203J, and RING finger protein genes, were reported to be highly induced during compatible and incompatible interactions with the pathogen (Chacón et al., 2009). On the contrary, functional analysis indicated that silencing of *GST* resulted in a significant increase in the resistance of plants to black shank disease as a result of upregulation of some defense genes (Hernández et al., 2009). These inconsistent results might sound reasonable because different *GST* genes were found to respond to fungal infection in different ways (Dean et al., 2005). Another type of defense-related genes includes serine/threonine protein kinase genes, which encode receptor proteins that mediate signal transduction in plant defense response. The overexpression of *NrSTK* significantly enhanced resistance to black shank in tobacco (Gao et al., 2015). In the present study, a novel category of the gene conferring resistance to black shank in tobacco was characterized as a cytochrome P450 gene, named as *SoCYP85A1*. Transgenic tobacco plants overexpressing *SoCYP85A1* showed enhanced resistance to *P. nicotianae*, although different transgenic lines exhibited different degrees of resistance to this pathogen. Among them, the L8 showed the highest resistance to black shank disease, which could result from the greatest induction at the transcriptional level. These results demonstrated that *SoCYP85A1* could participate in conferring resistance against *P. nicotianae* in plants.

### CS Is a Bioactive Component Which Is Induced in Tobacco Plants Upon *P. nicotianae* Infection

To dissect the elaborate biological function of *SoCYP85A1*, we overexpressed this gene in tobacco plants and examined the endogenous content of BRs. The result showed that most of the intermediates involved in the BR biosynthetic pathway were undetectable, with the exception of CS, which was accumulated remarkably. Similar results were obtained in tomato and poplar plants overexpressing *PtCYP85A3* (Jin et al., 2017). However, the endogenous level of CS in the *35S-CYP85A1* *Arabidopsis* was almost the same as that in wild-type, whereas high levels of 28-norCS, CS, and BL were detected in transgenic *Arabidopsis* plants overexpressing *AtCY85A2* (Kim et al., 2005). The dissimilarity could be explained by the fact that the CYP85 family members function uniquely in BR metabolism in different species. Taken together, *SoCYP85A1* might be a functional allele of *PtCYP85A3* and *AtCY85A2.*

In addition, transgenic plants overexpressing *SoCYP85A1* showed significant accumulation of CS, especially when subjected to *P. nicotianae* infection, which implied that CS might be a pathogen-infection-induced compound. Besides, the increased content of CS conferred enhancement of black shank resistance, indicating that CS might act as a bioactive component functioning in resistance to pathogen invasion. As another bioactive component among the BRs, BL is one of the most potent steroid hormones in plants. In *Arabidopsis*, an interplay between the plant immune system and BL was elucidated that BL biosynthesis could modulate the immunity of plants through BRI1-Associated Kinase 1 (BAK1) (Belkhadir et al., 2012). Therefore, BL was demonstrated to be involved in the response of *Arabidopsis* plants to pathogen attack. However, unexpectedly, in the present study, the BL content displayed no significant changes in both the WT and transgenic tobacco plants before and after pathogen treatment and rather CS was confirmed to be involved in the response of plants to infection. Regarding these inconsistencies, it appeared reasonable to assume that BRs might exert their biological effects through different intermediates or through the final product in different species. This assumption was also supported by the fact that 28-nor CS was identified as the bioactive compound in tomato (Li et al., 2016a).

### Alteration in the Content of CS Interferes With Homeostasis of Phytohormones

During the last decade, various phytohormones have been identified to be involved in the modulation of plant–pathogen interactions (Zhou et al., 2005; Fan et al., 2009; Vlot et al., 2009; De et al., 2012; Nagel et al., 2017). BRs are important phytohormones involved in various aspects of plant development (Kim et al., 2005; Li et al., 2016a,b). Recently, a number of evidences have indicated that BRs can also participate in the resistance of plants to pathogens (Albrecht et al., 2012; Belkhadir et al., 2012; Prince et al., 2014). However, the interplay between BRs and other phytohormones has remained unresolved.

In this research, we studied the interaction between CS and four other phytohormones (ABA, GA, JA, and SA) by taking advantage of the fact that the concentrations of CS are highly regulated in transgenic seedlings overexpressing *SoCYP85A1*, especially when attacked by *P. nicotianae*. In the absence of the pathogen, the contents of ABA, GA, and JA in transgenic seedlings displayed no obvious changes, compared to the levels in the WT plants. We speculated that this was because of the fact that although CS was significantly accumulated, this increase did not have an effect on the three phytohormones. When CS was accumulated up to a certain level, it was worth noting that the concentrations of the four hormones in transgenic plants were much less than those in the WT plants upon challenge with the pathogen. Based on the result, the antagonistic interaction between CS and the three hormones could not take place until its content reached a critical level; however, even lower content of CS could reduce the biosynthesis of SA. Therefore, upon pathogen challenge, CS could act antagonistically on ABA, GA, JA, and SA by upregulating the *CYP85A1* expression in tobacco plants. A similar interaction was observed between BRs and JA, wherein JA was shown to interrupt BR signaling by downregulating the expression of *CYP90B1* during the response of plants to pathogens (Kim et al., 2013).

### Activities of Antioxidant Enzymes Are Upregulated by *SoCYP85A1* at Transcriptional Level

To resist pathogen invasion, plants have evolved defense mechanisms, in which different enzymes play the most important roles (Lebeda et al., 2001). In our study, we observed that the activities of SOD, POD, PPO, and CAT were highly regulated in L8 compared to those in the WT plants, which indicated that transgenic lines possessed more efficient detoxification system against phytopathogen attack. It was reported that a combination of these antioxidant enzymes in plants might contribute to greater resistance of plants to the pathogens (Hafez et al., 2012; Leite et al., 2014).

To further explore the mechanism through which *SoCYP85A1* enhanced black shank resistance at the molecular level, qRT-PCR was conducted to examine the transcript levels of defense-related genes. The transgenic lines overexpressing *SoCYP85A1* exhibited significant induction of the four genes encoding antioxidant enzymes at the transcriptional level when attacked by the pathogen. These results correspond with those for enzyme activities, implying that the activities of antioxidant enzymes were remarkably increased because of upregulation of the transcription of related genes by *SoCYP85A1*. A similar mechanism was also revealed in a previous study that increased transcription of defense enzyme genes conferred resistance to *P. nicotianae* in transgenic tobacco plants (Hernández et al., 2009).

The pathogen attack, however, did not induce increased transcript accumulation of ABA biosynthetic gene (*NtNCED1*), SA-responsive genes (*NtPR1a/c* and *NtPR2*), JA-responsive gene (*NtPR1b*), and the HR-associated gene (*NtHSR203J*) in transgenic lines, in consonance with the results of ABA, JA, and SA quantification (Figure 4). This implied that the above genes were not transcriptionally upregulated by *SoCYP85A1* in tobacco plants. Further, we speculated that overexpression of *SoCYP85A1* enhanced black shank tolerance in an ABA-, JA-, and SA-independent manner.

Based on the above findings, we concluded that the enhanced resistance of transgenic plants to pathogen infiltration was a result of increased CS accumulation and improved activities of antioxidant enzymes, which were transcriptionally regulated by *SoCYP85A1* via an ABA, JA, and SA-independent pathway. However, although the influence of *SoCYP85A1* on pathogen resistance was studied in the present study, a comprehensive and elaborate elucidation of the regulatory mechanism underlying the defense response of these transgenic tobacco plants to pathogen invasion would require further investigation.

## Author Contributions

WS designed the research and wrote the manuscript. FD characterized the transgenic plants and conducted all the experiments.

## Conflict of Interest Statement

The authors declare that the research was conducted in the absence of any commercial or financial relationships that could be construed as a potential conflict of interest.
